# Validation of a Portable Blood Gas Analyzer for Use in Challenging Field Conditions at High Altitude

**DOI:** 10.3389/fphys.2020.600551

**Published:** 2021-01-08

**Authors:** Janek Nawrocki, Michael Furian, Aline Buergin, Laura Mayer, Simon Schneider, Maamed Mademilov, Madeleine S. Bloch, Talant M. Sooronbaev, Silvia Ulrich, Konrad E. Bloch

**Affiliations:** ^1^Department of Respiratory Medicine, Sleep Disorders Center, University Hospital of Zürich, Zurich, Switzerland; ^2^National Center of Cardiology and Internal Medicine, Bishkek, Kyrgyzstan

**Keywords:** blood gas analyzer, point-of-care testing, arterial blood gas (ABG) analyzers, hypoxaemia respiratory failure, hypercapnia, high altitude

## Abstract

**Background:**

Novel, portable blood gas analyzers (BGAs) may serve as essential point-of-care tools in remote regions, during air travel or in ambulance services but they have not been extensively validated.

**Research Question:**

We compared accuracy of a portable BGA to a validated stationary device.

**Methods:**

In healthy individuals and patients with chronic obstructive pulmonary disease participating in clinical field studies at different altitudes, arterial blood samples were obtained at rest and during exercise in a hospital at 760 m and in a high altitude clinic at 3100 m. Paired measurements by a portable BGA (EPOC, Siemens Healthcare) and a stationary BGA (Rapidpoint500, Siemens Healthcare) were performed to compute bias (mean difference) and limits of agreement (95% CI of bias).

**Results:**

Of 105 individuals, 248 arterial blood samples were analyzed, 108 at 760 m, 140 at 3100 m. Ranges of values measured by portable BGA were: pH 7.241−7.473, PaCO_2_ 21.5−52.5 mmHg, and PaO_2_ 45.5−107.1 mmHg. Bias (95% CI) between devices were: pH 0.007 (−0.029 to 0.044), PaCO_2_ −0.3 mmHg (−4.8 to 4.2), and PaO_2_ −0.2 mmHg (−9.1 to 4.7). For pH, agreement between devices was improved by the equation to correct pH by portable BGA = −1.37 + pH_*measured*_ × 1.19; bias after correction −0.007 (−0.023 to 0.009). The portable BGA was easily handled and worked reliably.

**Interpretation:**

Accuracy of blood gas analysis by the portable BGA in comparison to the reference BGA was adequate for clinical use. Because of portability and ease of handling, portable BGA are valuable diagnostic tools for use in everyday practice as well as under challenging field conditions.

## Introduction

Mountain travelers as well as airplane passengers experience hypobaric hypoxia that may induce hypoxia-related illness and changes in metabolic conditions. Patients with cardiovascular or pulmonary diseases are particularly susceptible. Unfortunately, there is a lack of information about these health risks, partly due to the heavy weight of conventional diagnostic instruments for biochemical analysis of blood, the required external electrical power supply, and related major logistical efforts and costs. To address these issues, novel, portable devices for the analysis of blood gases, electrolytes and other parameters have been developed.

The EPOC device (Siemens Healthcare) is a battery-operated, portable blood gas analyzer (BGA) for use in challenging point-of-care settings such as in remote mountain regions, during air travel, or in emergency ambulance services (Epocal Inc., 2020). Near sea level, the EPOC portable BGA has been evaluated in detail and proven to have high accuracy (Nichols et al., 2008; Nicolas et al., 2013; Stotler and Kratz, 2013; Luukkonen et al., 2016). The main advantages of the EPOC are its portability due to the low weight of only 500 g, battery driven operation, and wireless connectivity. In addition, the EPOC test cards can be stored at room temperature and do not need to be refrigerated. To what extent the EPOC allows accurate blood gas measurements in field conditions such as in high mountain areas still requires further investigations (Leino and Kurvinen, 2011).

The purpose of the current study was to comprehensively validate the EPOC in comparison to an established stationary reference device (Rapidpoint500, Siemens Healthcare) (Siemens Medical Solutions USA, 2020). We performed paired analyses of samples from healthy individuals and from patients with chronic obstructive pulmonary disease (COPD) participating in studies at different altitudes (760 and 3100 m) at rest and during exercise. This assured that arterial blood gases and pH could be studied over a wide range of values. We tested the hypothesis that the portable BGA (EPOC) measures arterial blood gases (pH, PaO_2_, and PaCO_2_) with clinically acceptable accuracy within ranges specified by the clinical laboratory improvement amendments (CLIAs) (Astles et al., 2013) compared to the stationary reference device (Rapidpoint500).

## Materials and Methods

### Study Design and Setting

In this study, arterial blood samples from participants in two randomized, placebo-controlled, double-blind, parallel trials were analyzed. The initial trials evaluated effects of preventive acetazolamide treatment (375 mg/day) on the incidence of acute mountain sickness (AMS) during a stay at 3100 m in patients with COPD and in age-matched healthy controls (Furian et al., 2019). The results of blood analysis by the EPOC device, the focus of the current study, has not been published. After baseline evaluations in the National Center of Cardiology and Internal Medicine (NCCIM) in Bishkek, Kyrgyzstan, 760 m, participants traveled by bus to a remote high-altitude clinic at 3100 m and stayed there for two nights. Over the course of the studies, repeated arterial blood samples were obtained at rest and during cycling exercise tests and analyzed by the EPOC and the reference device, the Rapidpoint500. The protocol was approved by the Ethics Committees of the NCCIM and participants provided written informed consent.

### Participants

In trial 1, men and women with COPD living below 800 m were recruited. Inclusion criteria were an age of 18–75 years, COPD diagnosed according to GOLD, FEV_1_ 40–80% predicted, pulse oximetry (SpO_2_) > 91%, and PaCO_2_ < 45 mmHg at 760 m. In trial 2, healthy individuals of the same age were admitted. The exclusion criteria were any acute disease or unstable health condition and allergy to sulfonamides.

### Measurements and Outcomes

Arterial blood samples were collected once in the morning and over the course of the day before and toward the end of an exercise test, both at 760 and 3100 m. Each sample was analyzed immediately in triplicate, first by the Rapidpoint500, then by the EPOC and, finally, again by the Rapidpoint500. The value from the EPOC device was compared to the mean of the corresponding values by Rapidpoint500 to avoid bias from a time delay between measurements by the two devices. Changes in variables over the course of successive measurements in the same person were also analyzed. Over the course of 25 days, the repeatability of the EPOC and the Rapidpoint500 devices was additionally checked daily by comparison to two different calibration solutions with acidic and alkalotic pH ranges.

Processing of samples was performed according to manufacturer’s guidelines with special attention to pre-analytical factors (e.g., timing and elimination of bubbles in the samples). Three main variables (pH, PaCO_2_, PaO_2_) and eight other variables (hematocrit [Hct], hemoglobin concentration [tHb], glucose, lactate, sodium, potassium, calcium, and chloride) were analyzed. The limits for the clinical acceptability are ±5 mmHg for PaCO_2_, ±3SD (±8.0 mmHg) for PaO_2_, and ±0.04 for pH according to CLIA (Astles et al., 2013).

### Devices

The EPOC is a battery powered device, dimensions 60 × 180 × 30 mm, weight 500 g, with a touch-screen display (25 × 30 mm), integrated barcode scanner, and optional external printer. According to the manufacturer, EPOC can be operated at barometric pressure equivalents of altitudes up to 5100 m and temperature 15–30°C. For blood gas analysis, a droplet of blood is inserted into a small cavity of a test card containing sensors for the analysis. The calibration and analysis take approximately 7 min. Results are shown on the display and can be transmitted by wireless (bluetooth) connection to a printer and to a computer as needed. The EPOC test cards can be stored at room temperature (15–30°C).

The Rapidpoint500 is a stationary BGA (dimensions 420 × 550 × 300 mm, weight 16.6 kg, with a touch-screen display 211 × 158 mm) with a turnaround time of 3–4 min. Cartridges for 250 or 500 measurements and waist are inserted into the device. Cartridges not inserted into the device require storage at 2–8°C. Operating conditions of the Rapidpoint500 are barometric pressure equivalents of altitudes up to 4572 m and temperature 15–30°C (Epocal Inc., 2020; Siemens Medical Solutions USA, 2020).

### Statistics

Data are summarized as means ± SD. Agreement and precision are quantified according to Bland and Altman and CLSI guidelines, reporting mean differences between methods as bias, and 95% confidence interval as limits of agreement (Giavarina, 2015). Trends of differences in agreement between methods were evaluated by linear regression analysis. A probability *P* < 0.05 was assumed as statistically significant.

## Results

In 105 individuals, 248 radial artery blood samples were obtained for paired analyses by both devices, 108 at 760 m and 140 at 3100 m. 119 samples were collected at rest and 129 samples during exercise. The mean time between the first and second measurement in Rapidpoint500 was 3 min and 10 s (2–19 min). EPOC measurements were taken in-between.

**FIGURE 1:**
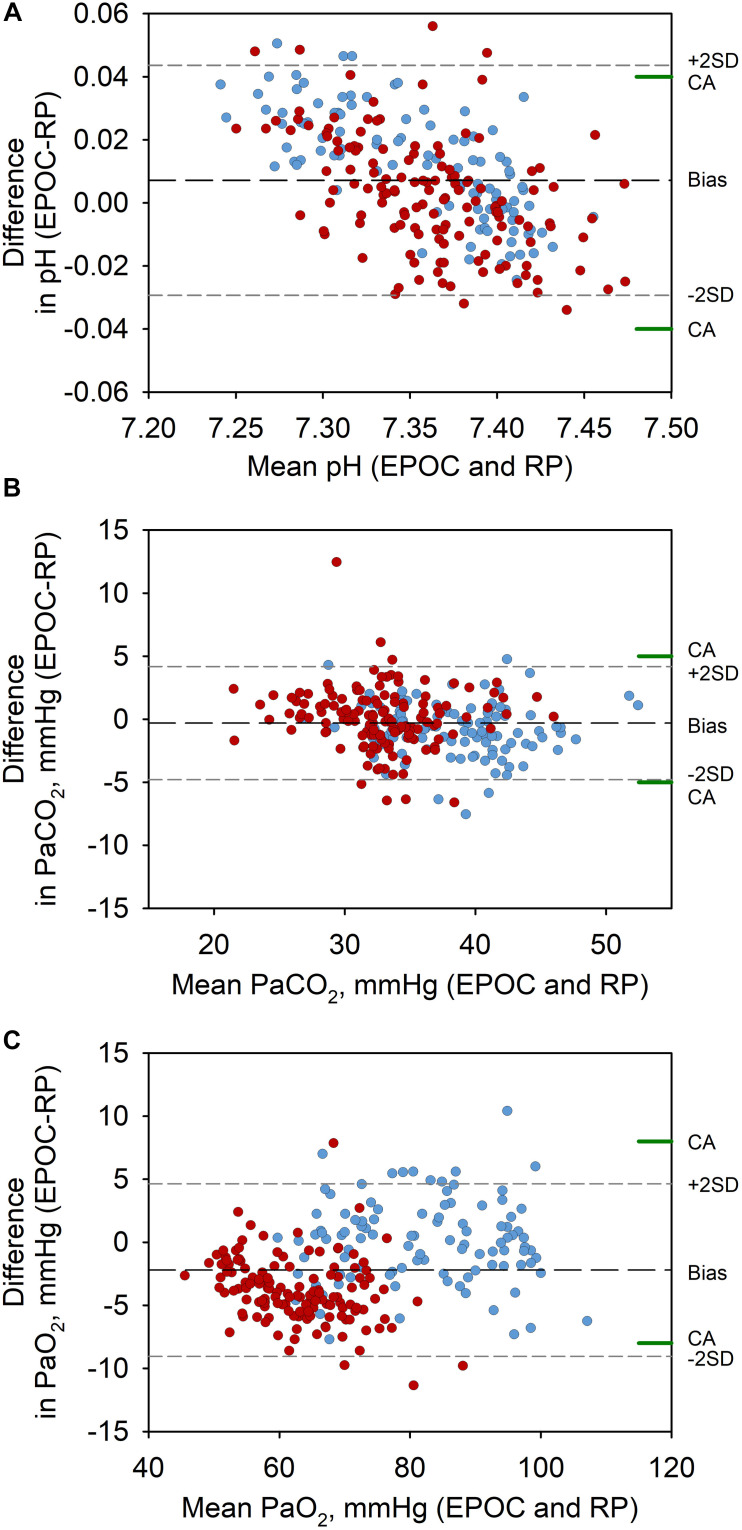
Bland-Altman plots for the comparison of the portable blood gas analyzer EPOC and the stationary reference device Rapidpoint500 in measurements of pH **(A)**, PaCO_2_ **(B)**, and PaO_2_ **(C)**. The *y*-axes represent the difference between values from corresponding measurements by EPOC and Rapidpoint500 (EPOC-RP), and the *x*-axes the mean of the values from the two devices. The mean difference (bias, long dashed line), the limits of agreement (dashed, gray lines marked ± 2SD) and the clinical acceptability range according to CLIA (Astles et al., 2013) (range between green lines labeled CA on the right *y*-axis) are shown. Blue and red symbols represent values obtained at 760 and 3100 m, respectively.

**FIGURE 2:**
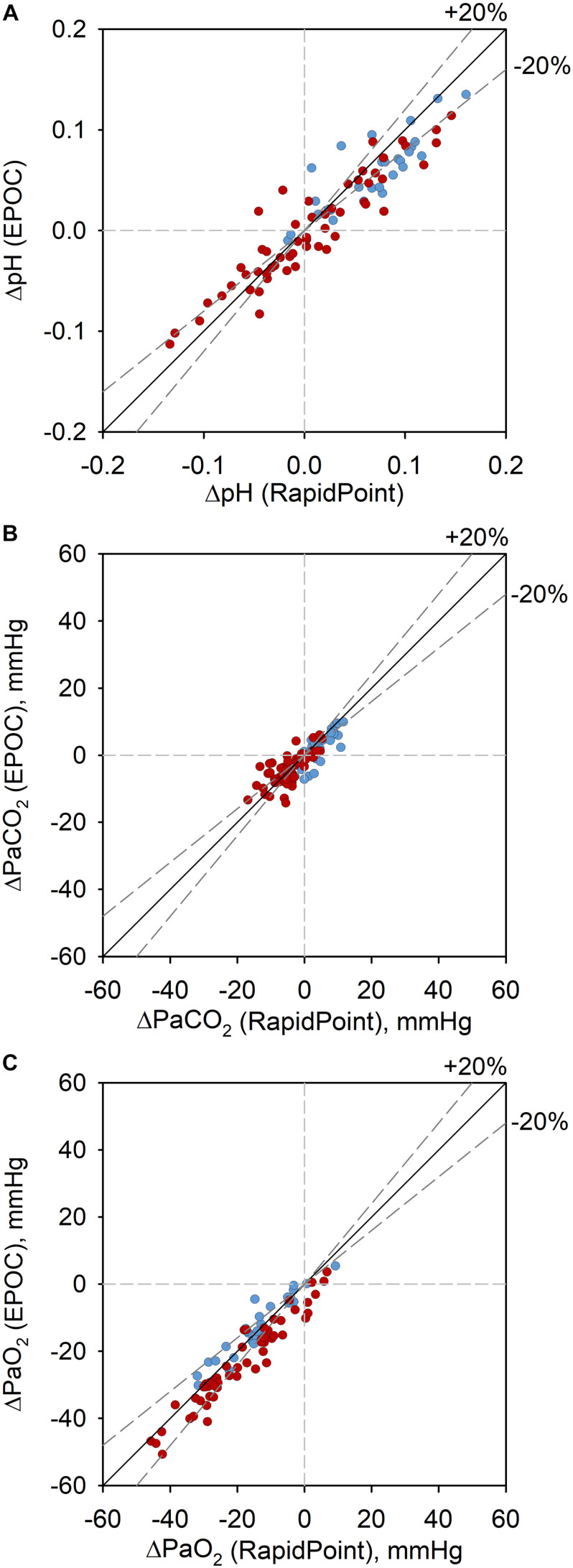
Identity plots of intra-individual changes in pH **(A)**, PaCO_2_ **(B)**, and PaO_2_ **(C)** (ΔpH, ΔPaCO_2_, and ΔPaO_2_) measured by EPOC and Rapidpoint500 in 14 patients over the course of studies at 760 and 3100 m at rest and at peak exercise. Identity lines and ±20% deviation from identity (dashed, gray lines) are shown. Intra-individual changes observed at the same altitude (760 and 3100 m) are represented by blue symbols, changes between 760 and 3100 m are represented by red symbols.

The comparison of blood gases between EPOC and Rapidpoint500 are summarized in Table 1 and illustrated in Figure 1. There was good agreement between the paired measurements without a significant bias. The proportions of values measured by EPOC that fell within the range of acceptability compared to values from Rapidpoint500 were 96% for pH, 96% for PaCO_2_, and 97% for PaO_2_. The 95% confidence interval for pH (−0.029 to 0.044) was only in the lower range within the clinical acceptability of ±0.04. The 95% CI for PaCO_2_ (−4.8 to 4.2 mmHg) was within the clinical acceptability range of ±5.0 mmHg. The 95% confidence interval for PaO_2_ (−9.1 to 4.7 mmHg) was only in the upper range within the clinical acceptability range of ±8 mmHg. Figure 2 shows identity plots of changes in pH and blood gases over the course of the study in 14 participants.

**TABLE 1:** Agreement among EPOC and Rapidpoint500 measurements.

Variable	Rapidpoint500	EPOC	Mean difference ± SD (EPOC – Rapidpoint500)	Limits of agreement (95% CI)	Clinical acceptability
pH	7.355 ± 0.055	7.362 ± 0.044	0.007 ± 0.019	−0.029 to 0.044	±0.04
PaCO_2_ (mmHg)	35.4 ± 5.6	35.1 ± 5.2	−0.3 ± 2.3	−4.8 to 4.2	±5.0
PaO_2_ (mmHg)	72.1 ± 13.1	69.9 ± 14.2	−2.2 ± 3.5	−9.1 to 4.7	±3SD, ±8.0

*Values are presented as mean ± SD. The suggested clinical acceptability range is obtained from the Astles et al. (2013), CLIA requirements for proficiency testing.*

Regression analysis of values measured by EPOC as dependent and values measured by Rapidpoint500 as independent variables provided the following equation to correct the PaO_2_: PaO_2__*corrected*_ = 9.45 + PaO_2__*measured*_ × 0.90 (*R*^2^ = 0.94), corrected bias 0 mmHg (−6.2 to 6.2). A trend for overestimation of pH by EPOC in the low and underestimation in the high range could be corrected by the equation pH_*corrected*_ = −1.37 + pH_*measured*_ × 1.19 (*R*^2^ = 0.91), corrected bias −0.007 (−0.023 to 0.009). No significant trend could be detected for PaCO_2_ (*R*^2^ of regression for PaCO_2_ = 0.7).

To test repeatability of measurements, we performed repeated measurements of standard reference solutions with EPOC (total of 54) and Rapidpoint500 (*n* = 50) over a period of 25 days (Table 2). The ranges in repeated measurements with two reference solutions (acid and basic) measured by EPOC were: pH 6.92–7.61, PaCO_2_ 17.6–86.6 mmHg, and PaO_2_ 48.4–256.6 mmHg. Coefficients of variation (SD/mean) for the results in EPOC were for pH 0.2%, PaCO_2_ 3.6%, and PaO_2_ 7.2%. The ranges in Rapidpoint500 were pH 7.136–7.584, PaCO_2_ 20.7–75.3 mmHg, and PaO_2_ 61.7–176.2 mmHg. The coefficients of variation for the Rapidpoint500 were pH 0.04%, PaCO_2_ 1.8%, and PaO_2_ 1.3%.

**TABLE 2:** Repeatability of pH and blood gas measurements.

Variable	Mean ± SD	Coefficient of variation (%)	Observed range
pH EPOC	7.265 ± 0.014	0.2	6.918–7.610
pH Rapidpoint500	7.348 ± 0.003	0.04	7.136–7.584
PaCO_2_ EPOC (mmHg)	51.3 ± 1.9	3.6	17.6–86.6
PaCO_2_ Rapidpoint500 (mmHg)	46.2 ± 0.9	1.8	20.7–75.3
PaO_2_ EPOC (mmHg)	125.3 ± 9.1	7.2	48.4–256.6
PaO_2_ Rapidpoint500 (mmHg)	110.8 ± 1.5	1.3	61.7–176.2

*Values are presented as mean ± SD, *n* = for EPOC, and *n* = for Rapidpoint500. Coefficients of variation are computed as SD/mean of two consecutive measurements by the same blood gas analyzer.*

Handling of EPOC was easy and the device worked reliably. When the test cards were used according to the guidelines, they did not have any failures. Calibration of each test card took 3 min, the analysis time for one measurement was maximally 7 min. The data were transmitted quickly from the reader to the computer or a printer via bluetooth connection. During 248 measurements with EPOC no problems with the technique or the software were encountered. The test cards, stored at room temperature, worked flawlessly.

For the sake of completeness and future reference, exploratory comparisons of additional variables have also been performed and are reported in Table 3.

**TABLE 3:** Comparison of additional variables between EPOC and Rapidpoint500.

Variable	Rapidpoint500	EPOC	Range	Mean difference	95% CI	Clinical acceptability
Hct (%)	45.0 ± 4.6	44.0 ± 5.5	26.3–57.3	–0.99	−5.44 to 3.45	±6%
tHb (g/dl)	15.3 ± 1.6	15.0 ± 1.8	9.0–19.4	–0.33	−1.84 to 1.18	±7%, ±1.07
Glu (mmol/l)	5.84 ± 1.34	5.91 ± 1.35	3.20–14.28	0.07	−0.48 to 0.61	±10%, ±0.58
Lac (mmol/l)	4.56 ± 3.77	4.73 ± 4.26	0.47–14.48	0.17	−1.22 to 1.56	±20%, ±0.91
Na (mmol/l)	142.2 ± 3.2	143.8 ± 2.7	137.1–151.2	1.54	−2.03 to 5.11	±4.0
K (mmol/l)	4.19 ± 0.53	4.07 ± 0.48	3.29–5.78	–0.12	−0.33 to 0.09	±0.5
Ca (mmol/l)	1.24 ± 0.05	1.22 ± 0.05	1.07–1.33	–0.01	−0.09 to 0.06	±0.25
Cl (mmol/l)	109.0 ± 2.5	109.8 ± 2.9	104.0–117.8	0.80	−2.40 to 3.99	±5%, ±5.5

*Values are presented as mean ± SD. The recommended clinical acceptability range is obtained from the Astles et al. (2013), CLIA requirements for proficiency testing.*

## Discussion

In the current study, we validated the new portable BGA EPOC in comparison to the established stationary device Rapidpoint500. We found close agreement between the two devices in measurement of the pH, PaCO_2_, and PaO_2_ with ≥96% of values measured by the EPOC falling within the predefined range of clinically acceptable accuracy compared to the reference (Astles et al., 2013). If linear regression analysis was applied to correct for minor trends of differences between devices near perfect agreement could be obtained, i.e., all measurements were within the clinically acceptable range. In our study, including measurements in a hospital setting and in a high-altitude clinic with measurements performed at rest and during exercise, the EPOC was easy to use and reliable. Therefore, our study suggests that the device is a valuable tool for application in various, challenging field conditions, specifically including high altitude.

Previous studies have evaluated the EPOC near sea level. Luukkonen et al. (2016) compared the EPOC with Rapidpoint500 and three other stationary BGA. Among 72 critically ill patients, there was good correlation between devices (*R*^2^ > 0.96) for the pH and PaO_2_ but *R*^2^ = 0.87 for PaCO_2_ was slightly reduced with a significant bias and limits of agreement exceeding the recommended range. Unfortunately, a formal analysis of bias and precision of the measurements in absolute units was not presented which hampers a direct comparison to the current results. Nichols et al. (2008) analyzed 143 blood samples at five different locations with the EPOC and another portable BGA, the i-STAT (Abbott Point of Care). The EPOC showed a superior repeatability than the i-STAT for pH and blood gases (coefficient of repeatability of 0.08–3.0% vs. 0.17–5.3%) but the statistical analysis was not conclusive in terms of agreement between devices as no bias and limits of agreement were reported. Nevertheless, the authors pointed out the benefits of the EPOC test cards that could be stored at room temperature without the need of a refrigerator which facilitates logistics in a point of care setting. Chen et al. (2016a) analyzed 118 specimens from 40 patients undergoing cardiopulmonary bypass surgery using the stationary device GEM4000 (Instrumentation Laboratory), 3 EPOC devices, and 2 other BGA. Bias between EPOC and GEM4000 were for pH −0.004, PaCO_2_ −0.9 mmHg, and PaO_2_ 6.4 mmHg. Among the two point-of-care devices, EPOC and i-STAT, they found bias’s in pH 0.001, PaCO_2_ −2.3 mmHg, and PaO_2_ 13.3 mmHg which were greater than the corresponding bias’s among EPOC and Rapidpoint500 in the current study (PaCO_2_ −0.3 mmHg and PaO_2_ 2.2 mmHg). In the same data set (Chen et al., 2016b), a linearity analysis of pH, PaCO_2_, and PaO_2_ revealed values of *R*^2^ > 0.95 for EPOC vs. GEM4000, i-STAT and NovaCCX (Nova Biomedical). Stotler and Kratz (2013) reported considerable bias among EPOC, Nova CXX, and i-STAT in point-of-care settings and the EPOC tests had a relatively high failure rate in the first year after implementation in hospitals. During our investigations, we had no failures with EPOC analyses that were performed strictly according to manufacturer’s guidelines by well-trained users suggesting that this was essential for successful application.

In two studies, results of capillary blood analyzed with EPOC were compared to corresponding results of venous or arterial samples performed with standard devices in critically ill patients (Kim and Kim, 2018; Shin et al., 2020). The studies suggested that portable BGA are useful for application in intensive care or emergency services when rapid sampling and on-site analysis of blood is essential.

Further studies performed with blood samples from animals (horses or dogs) are not discussed in detail here. These results showed a good agreement between EPOC and the reference analyzers (Elmeshreghi et al., 2018; Kirsch et al., 2019).

A major strength of our study was the use in various settings, i.e., at low and high altitude, in a large tertiary care center and in a remote high-altitude clinic, with a wide range of variables measured due to the testing at rest as well as during exercise. A limitation was that we compared the EPOC to only one type of stationary BGA even though the Rapidpoint500 is a well validated device. The storage of EPOC test cards at room temperature without dependency on a refrigerator was an important advantage for application of the device under field conditions. A limitation of the EPOC was a relatively long calibration time of 3 min after insertion of the test card. While this might be adequate for point-of-care assessments in an individual patient it might represent a limitation in a hospital setting with high-volume measurement sequences.

## Conclusion

The results of the current study show that EPOC allows accurate measurements of arterial blood gases in a challenging field setting. As the device was easy to use and robust it might serve as a valuable tool for point-of-care applications including at high altitude, during transports and in remote locations where electrical power is not available.

## Data Availability Statement

The datasets presented in this article are not readily available because Data will be made available for research purposes within limits set by the ethics committee. Requests to access the datasets should be directed to KB.

## Ethics Statement

The studies involving human participants were reviewed and approved by Ethics Committee National Center of Cardiology and Internal Medicine, Bishkek, Kyrgyzstan. The patients/participants provided their written informed consent to participate in this study.

## Author Contributions

KB, AB, and MF contributed to the conception and design of the study, data collection, analysis, interpretation, and drafting the article. LM, SS, MM, and MB contributed to data collection, analysis, and interpretation of data. TS, SU, and KB contributed to obtaining funding, to the conception and design of the study, acquisition, analysis, and interpretation of data. All authors critically revised the manuscript for important intellectual content, they approved the version to be published and all agree to be accountable for all aspects of the work in ensuring that questions related to the accuracy or integrity of any part of the work are appropriately investigated and resolved. All authors contributed to the article and approved the submitted version.

## Conflict of Interest

KB reports grants to his institution from the Swiss National Science Foundation. The remaining authors declare that the research was conducted in the absence of any commercial or financial relationships that could be construed as a potential conflict of interest.
